# A Companion App to Support Rheumatology Patients Treated with Certolizumab Pegol: Results From a Usability Study

**DOI:** 10.2196/17373

**Published:** 2020-07-27

**Authors:** Barbara Domańska, Stijn Vansant, Irina Mountian

**Affiliations:** 1 UCB Pharma Slough United Kingdom; 2 UCB Pharma Brussels Belgium

**Keywords:** rheumatology, internet, digital health, mobile health, mHealth, smartphone, mobile phone, validation human factors study

## Abstract

**Background:**

Certolizumab pegol (CZP) is an anti-tumor necrosis factor drug approved for the treatment of multiple moderate to severe chronic inflammatory diseases. In the European Union, CZP is approved for administration by subcutaneous self-injection using a prefilled syringe, prefilled pen, or reusable electromechanical auto-injector (electronic device). CimplyMe is a companion app for use alongside CZP self-injection devices, designed to support CZP-treated patients self-managing their treatment and disease.

**Objective:**

This study aimed to validate the usability of the companion app by demonstrating that tasks required for use can be performed successfully by intended end users.

**Methods:**

We recruited 15 patients with moderate to severe rheumatoid arthritis, currently prescribed biologic treatment, and using apps on a smart phone. Patients were assessed on their ability to use the companion app in a setting designed to simulate a location where patients regularly administer biologic treatment. To assess the usability of the key features of the app, 8 critical and 3 noncritical tasks were designed. Patients’ success on each task was recorded through observations or knowledge-based questions. Successes with difficulty and use errors were also recorded. If a patient made a use error at the first attempt, a second attempt was allowed. Second-attempt use errors were recorded as a task failure.

**Results:**

A total of 207 first attempts at the 14 components of the 8 critical tasks were evaluated (3 patients failed to complete one component); 178 (86.0%) critical tasks were successfully completed at the first attempt. The remaining first attempts comprised 16 (7.7%) successes with difficulty and 13 (6.3%) use errors, which had to be repeated. One critical task was not re-attempted by one patient due to time constraints; however, there were no use errors in the 12 completed second attempts. A total of 107 first attempts at the 3 noncritical tasks were made, all of which (107/107, 100.0%) were completed without use errors.

**Conclusions:**

In simulated testing, patients were able to successfully use the companion app without formal training. This study suggests the companion app is easy to use and could help patients prescribed CZP better manage their treatment and disease.

## Introduction

Anti-tumor necrosis factors (TNFs) are established and effective treatments for moderate to severe chronic inflammatory diseases [1]. The use of anti-TNFs alongside conventional disease-modifying antirheumatic drugs has been proven to result in better long-term disease control and reduced functional impairment [1].

Certolizumab pegol (CZP) is an Fc-free, PEGylated anti-TNF approved for use in 66 countries [2]. CZP is approved to treat adults with moderate to severe rheumatoid arthritis (RA), axial spondyloarthritis (including both radiographic and nonradiographic axial spondyloarthritis), psoriatic arthritis, and plaque psoriasis in European Union (EU) countries and is also indicated for Crohn’s disease in the United States [3,4]. In the EU, CZP treatment is administered via prefilled syringe or prefilled pen [5]. Recently, an electromechanical autoinjection device (electronic device [e-Device]), ava, has also been approved for use [3,5]. All CZP-injection devices were designed with patient input. The e-Device aims to provide patients with customizable features to improve patient satisfaction and overall self-injection experience [5].

Subcutaneous self-injection is associated with several specific challenges that can lead to reduced treatment adherence. Increasing use of smartphones and tablets provides a unique opportunity to address some of these challenges. For example, mobile phone apps can be designed to support patients and provide a means to overcome challenges such as forgetfulness [6]. Previous studies looking into the effectiveness of mobile phone health apps suggest that if well designed, they may provide an effective tool, empowering patients with long-term chronic conditions to self-manage their own health [7-9]. CimplyMe is a new mobile technology companion app designed to be used alongside any CZP self-injection device to further support and engage CZP-treated patients throughout their disease journey. By providing additional support, the companion app aims to improve patient satisfaction, treatment adherence, and resulting clinical outcomes.

The aim of this study was to validate the usability of the companion app by demonstrating that critical tasks can be performed successfully by a group of patients diagnosed with RA, representative of the intended end users.

## Methods

### Study Design

This study assessed the usability of CimplyMe, a new mobile technology companion app designed to be used alongside any CZP self-injection device to further support and engage CZP-treated patients throughout their disease journey. The app provides step-by-step guidance through its timeline feature, providing information on the disease and CZP treatment. Guidance is focused on the initial 12 weeks of treatment but continues to support patients throughout their disease journey. Patients can track their pain, energy levels, mood, and activities using the Health Metrics Tracking feature. This is incorporated into the Health Summary that provides patients with an overview of their progress, including treatment adherence tracking, which they can choose to share with health care practitioners and their family. Milestones are included in the app and were designed to help patients acknowledge important moments in their disease journey and motivate patients to continue treatment. Additionally, injection and clinic appointment reminders aim to improve patient outcomes by supporting treatment adherence and disease monitoring.

**Figure 1:**
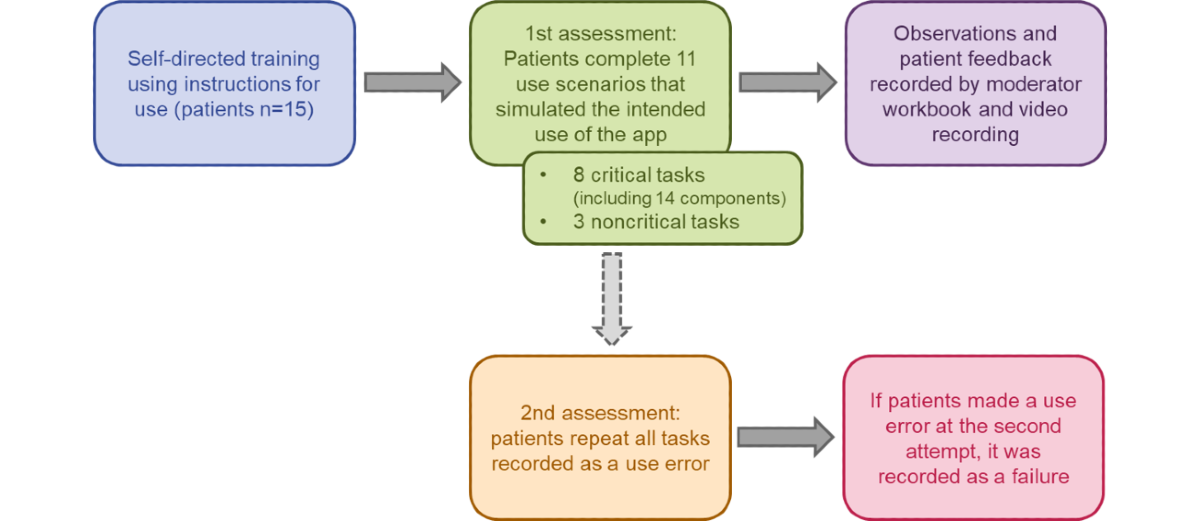
Study design flowchart.

This study was designed in line with ANSI/AAMI/IEC62366-1:2015 on applying human factors and usability engineering to medical devices [10]. Each participant gave informed consent before attending a single session; all sessions took place in London, United Kingdom. Figure 1 shows the study design. Patients were not given any formal training but had some time to familiarize themselves with the app. They were also able to use an instruction manual if requested, although the app was designed to be used without this document. Patients were asked to complete 11 use scenarios that were designed to simulate the intended use of the app. A moderator asked all patients to behave in the way they thought they would if the scenario was real. During the session, a moderator recorded all observations and patient feedback in a workbook. The sessions were also video recorded.

The primary study objective was to validate that the user interface of CimplyMe is intuitive, easy, and safe to use by intended users so as not to cause injury or harm. The secondary objective was to confirm that the risk of errors was reduced as far as reasonably possible through risk-mitigation methods implemented in the user interface (UI) of the beta version of the app used in this study.

### Study Procedures and Evaluations

Patients were assessed on their ability to complete 8 critical tasks composed of 14 component tasks and 3 noncritical tasks composed of 8 component tasks (Table 1). All participants were observed and timed while completing each task by study personnel. Each test session was scheduled to last for 105 minutes (1.75 hours); the moderator divided the time allocated between each task (between approximately 3 and 10 minutes depending on the task) to ensure that the participants could at least attempt each task once. Each component task had to be completed correctly; otherwise, a use error was recorded. If a patient struggled to complete the component tasks but avoided a use error, the task was recorded as a “success with difficulty.” If a patient made a use error on their first attempt, they could attempt the task a second time. If a use error was made at the second attempt, it was recorded as a task failure. All patient actions that led to, or resulted from, successes with difficulty or use errors were recorded. Detailed root cause analysis was carried out on all tasks completed with difficulty or recorded as a use error to determine whether the cause was a result of the user interface.

**Table 1:** Critical and noncritical tasks.

Tasks		Use scenarios
**Critical tasks**		
	1. Download companion app	Patients were asked to explain how they would go about downloading an app.
	2. Jailbroken phone^a^	Patients were shown the app terms and conditions and asked to explain what the message means. They were then asked if the app can be used on a jailbroken phone.
	3. Set up user profile (change language)	Patients were asked to change the language of the app.
	4. Set and adjust medication reminder	Patients were asked to explain how they would set up medication reminders. They were asked to schedule an injection reminder and edit it, following instructions. Finally, they were asked to delete the injection reminder.
	5. Manually enter injection dates	Patients were told to imagine they wanted to perform their injection ahead of schedule. They were given the date and locations of the injection they had just made and were asked to register the injection in the app.
	6. Log CZP^b^ injection date	Patients were asked to log an injection date that was outside of the logging time frame and asked if they understood the confirmation screen informing them that CZP was not logged within the correct time frame.
	7. Add or edit a CZP medication schedule	Patients were asked to imagine their treatment plan has changed and asked to change their dose regimen.
	8. Download app updates	Patients were asked what they would do if they opened the app and an “Updates Available” screen appeared.
**Noncritical tasks**		
	1. Edit or delete clinical appointments	Patients were asked to imagine they wanted to schedule a physician appointment and given information to input. They were then asked to edit the information they just inputted and finally to delete the reminder.
	2. Error messages	Several error messages from the app were presented. The patient was asked to explain what they thought the different messages mean.
	3. Remove profile from the app	Patients were asked to remove their user profile from the app.

^a^A jailbroken phone is one that has bypassed restrictions to allow the user to download apps from websites other than the official app store.

^b^CZP: certolizumab pegol.

### Participants

Adult patients (≥18 years) diagnosed with moderate to severe RA were invited to participate in the study. To ensure at least 97% of potential use errors were identified [11], a minimum of 15 intended users were recruited. To verify their RA diagnosis, patients were asked to provide a confirmation letter from their physician. In addition, patients had to have been prescribed biologic treatment at the time of enrollment. Patients must have been using a smart phone and had experience using apps, of any type, on their phone. Patients must also have stated that they would use a medical mobile app to assist with their disease treatment. The study aimed to recruit at least 7 patients with impaired vision, either correctable or not correctable with glasses or contact lenses.

Patients were excluded if they had participated in medical device research for a medical app in the previous 6 months, were app developers, or had stated that they would not use a mobile app to assist with their disease treatment. Patients were also excluded if they experienced <10 minutes of joint stiffness in the morning without treatment.

## Results

### Patient Disposition and Baseline Characteristics

Of the 30 patients who were screened to take part in the study, 15 patients completed the study. Of the 15 patients that did not take part, 8 were unable to provide a physician’s letter, 4 were not able to take part due to scheduling, and 2 patients self-withdrew. One patient was excluded as they were known to the sponsor. Familiarity with the use of mobile apps was a stated requirement; therefore, there were no screen failures relating to this.

Patient baseline characteristics are shown in Table 2. The mean patient age was 53.5 years (SD 12.0 years), and 66.7% (10/15) patients were female. All patients were using either a prefilled syringe or prefilled pen to administer their current biologic medication.

**Table 2:** Patient baseline characteristics.

Parameter		RA^a^ patients (n=15)
Age (years), mean (SD)		53.5 (12.0)
Gender (female), n (%)		10 (66.7)
**Ethnicity, n (%)**		
	White British	11 (73.3)
	Black British	2 (13.3)
	British Asian	2 (13.3)
Dominant hand (right), n (%)		14 (93.3)
**Highest education level achieved, n (%)**		
	Early years	0
	Primary	0
	Secondary	4 (26.7)
	A-Level	1 (6.7)
	College/university	6 (40.0)
	Postgraduate/vocational	4 (26.7)
**Vision (self-reported), n (%)**		
	Nearly perfect	4 (26.7)
	Need glasses to read	3 (20.0)
	Need glasses to see into the distance	2 (13.3)
	Need glasses to read and see into the distance	6 (40.0)
	Impaired vision and not correctable with glasses	0
Disease severity (moderate to severe), n (%)^b^		15 (100.0)
**Morning joint stiffness (minutes in the morning), n (%)**		
	0^c^	1 (6.7)
	≤30	4 (26.7)
	30-60	6 (40.0)
	>60	4 (26.7)
**Joints affected by RA, n (%)**		
	Fingers	12 (80.0)
	Hands	11 (73.3)
	Wrists	8 (53.3)
	Elbow	5 (33.3)
**Current biologic medication, n (%)**		
	Etanercept	6 (40.0)
	Certolizumab pegol	2 (13.3)
	Adalimumab	6 (40.0)
	Tocilizumab	1 (6.7)
**Type of self-injection device used, n (%)**		
	PFS^d^	7 (46.7)
	PFP^e^	8 (53.3)

^a^RA: rheumatoid arthritis.

^b^Physician-determined.

^c^When medicated; when unmedicated, stiffness ≥10 mins.

^d^PFS: prefilled syringe.

^e^PFP: prefilled pen.

### Critical Task Successes and Use Errors

Throughout the study, 207 first attempts at critical component tasks were completed. Of these, 86.0% (178/207) tasks were successful; 7.7% (16/207) were successes with difficulty; and 6.3% (13/207) were use errors (Table 3). Twelve second attempts at critical tasks were completed in the study; one task was not re-attempted due to time constraints. No use errors were encountered when performing tasks for a second time, although 4 tasks were recorded as successes with difficulty (Table 3).

Reasons for patient difficulties and use errors during first and second attempts at critical tasks are shown in Multimedia Appendix 1. Several (6/13, 46.2%) first attempts classed as use errors were cases of patients not completing the task in the allotted session time. Patients were not given a set amount of time to complete each task; however, the total time of each session was limited to 110 minutes so not all patients were able to complete all tasks within this time frame.

Difficulties at the first attempt were due to various reasons; however, most (10/16, 62.5%) resulted from patients getting confused by the app terms, organization, or process (Multimedia Appendix 1). Additionally, one difficulty was caused by an app fault. There were 4 successes with difficulty during the 12 second attempts at the critical tasks. As with those observed at first attempt, most (3/4, 75.0%) of these difficulties were due to patients getting confused by the app terms or organization (Multimedia Appendix 1).

**Table 3:** Critical and noncritical task outcomes.

Task and attempt		Success	Success with difficulty	Use error
**Critical tasks**				
	First (n=207)	178	16	13
	Second (n=12)^a^	8	4	0
**Noncritical tasks**				
	First (n=107)	104	3	0

^a^Only 12 patients re-attempted critical task 6, “log an injection date,” due to time constraints.

### Root Cause Analysis of Critical Task Errors

The root cause analysis of all use errors is shown in Table 4. The UI contributed to 61.5% (8/13) of use errors. Of these, 42.9% (3/8) were attributable to the small font size in the instructions for use (IFU). In 37.5% (3/8) of the use errors, patients did not see or were not prompted to scroll to the necessary button(s). The remaining errors associated with the UI (2/8, 25.0%) were attributed to app complexity or lack of clarity.

**Table 4:** Root cause analysis of all use errors.

Task and patient actions		Root cause	Did the UI^a^ contribute to the use error?
**4. Set and adjust medication reminder**			
	P09: did not save the reminder; opened IFU^b^ but did not read it after realizing it was 120 pages	Perception/cognition: expected the app to have a more assistive role in setting up a reminder	Yes: expected the app to do more and did not read the IFU
	P02: tried to edit the reminder but deleted it; confused tracking reminder and injection reminder	Cognition: did not understand what “Tracking Reminder” meant	Yes: unsure what tracking reminder meant
**5. Manually enter injection dates**			
	P03: did not register second injection	Perception/cognition: did not see the “Add Another” button and thought they would add both injections to the same image	No
	P04: took a long time to understand the task	Perception/cognition: could not see or understand where to register an injection	No
	P05: only registered one injection	Perception: did not see the “Add Another” button	Yes: did not see the “Add Another” button, only “Done”
	P06: kept trying to register injections through health tracking	Cognition: did not know where to go to register injections	No
	P10: did not scroll down to see “register injection”	Perception/cognition: did not see the button and did not think to continue scrolling	Yes: “Register Injection” was not visible on the screen, and there was nothing on the screen to suggest scrolling down
	P12: registered an injection but could not register a second injection	Cognition: expected to add both injections on the same image	Yes: text was very small in the IFU, which led to the patient skimming the text
**7. Add or edit a CZP^c^ medication schedule**			
	P02: expected “Change Treatment Plan” to be on the timeline	Cognition: thought they were looking in the obvious place to look	No
	P04: took a long time to complete the task	Cognition: struggled with the terminology of the app	No: patient struggled with the terminology throughout
	P09: expected to edit their dose regimen through the calendar	Perception: did not see the section in profile	No
	P11: not able to find where to edit the medication schedule	Perception/cognition: not able to find where to complete the task	Yes: patient said the complexity of the app made it more difficult
	P04: thought the app was faulty when the confirmation screen appeared	Cognition: patient found it difficult to understand the language and reasoning behind the confirmation screen	Yes: text could have been bigger and use of colors clearer

^a^UI: user interface.

^b^IFU: instructions for use.

^c^CZP: certolizumab pegol.

### Noncritical Task Successes and Use Errors

**Figure 2:**
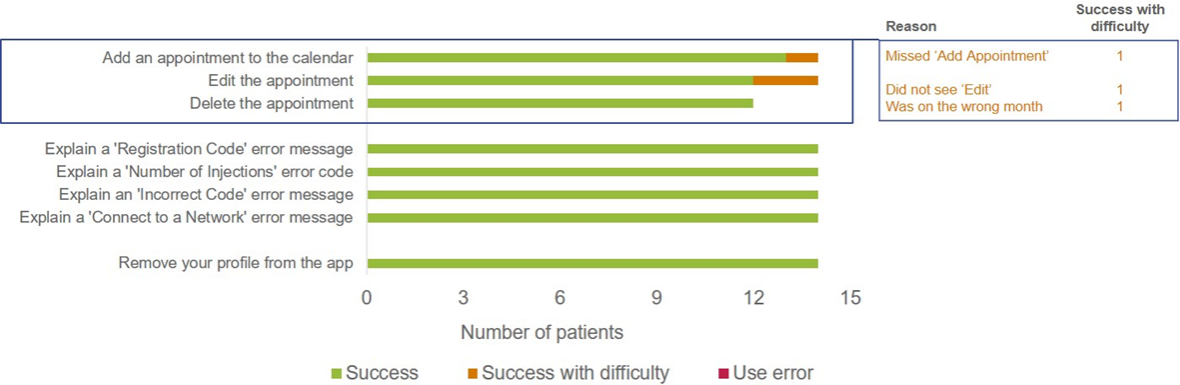
Reasons for noncritical task difficulties and use errors at first attempt. The number of patients who completed noncritical tasks was <15 as some patients did not have time to complete all tasks during the session.

A total of 107 first attempts at the 3 noncritical tasks were performed in the study. Of these, 97.2% (104/107) were successful, 2.8% (3/107) were successes with difficulty, and none were use errors (Table 3). Reasons for patient difficulties when completing noncritical tasks are shown in Figure 2.

## Discussion

### Principal Findings

No failures were observed for any of the attempted tasks; all patients were able to successfully complete both critical and noncritical tasks using the companion app on their first or second attempt without any formal training.

There were several instances where use errors could be attributed to the UI; these can all be resolved by small changes to the app. For example, patients did not realize that they needed to scroll further down the page to find the correct information or buttons in multiple instances. To minimize this problem, the scaling of the app could be optimized to ensure that most buttons are always on the screen. If future feedback suggests patients still have difficulties, a small note or animation could be added to indicate the need to scroll down on first use. Additionally, patients found the font size in the IFU and app too small or did not read the IFU because it was too long. However, the font size on the app is dependent on the default font size settings on the patient’s phone and can be changed by the patient, if necessary. Two patients did not understand the terminology used in the app, or struggled with the complexity of the app itself, resulting in confusion and error in completing tasks and navigating the app. However, this was likely due to the short amount of time patients were given to familiarize themselves with the app. It is anticipated that patient understanding of the app’s terminology and layout would improve with use, as indicated by the reduction in errors when tasks were attempted for a second time in this study.

Mobile technology apps have been developed to provide information and patient education, facilitate lifestyle changes as a strategy to ameliorate disease, provide medication reminders to promote treatment adherence, and record patient metrics for disease monitoring [12-15]. Previous research suggests that mobile technology apps can help patients feel supported and improve patient satisfaction [12,16]. For example, compared to usual care, a mobile health technology–supported disease management model for atrial fibrillation resulted in improvements in patient quality of life [17]. Patient adherence and disease management may also be improved with the support of treatment management apps [16,18,19]. For example, a 2019 systematic literature review of studies investigating mobile apps as a method to improve treatment adherence identified 11 studies of patients with a variety of diseases [18]. Of these, 7 studies reported an increase in treatment adherence with use of a mobile app [18].

One of the key challenges associated with the use of mobile apps is the high user attrition rate, with the level of mobile app engagement dropping off over time [20]. Understanding the features and characteristics most valued by patients may improve app engagement. Users appreciate features that save time over current methods and identify an app as valuable when it is simple and intuitive to use, provides specific instructions to better manage a condition, shares data with designated individuals, and incorporates motivational “push” factors [20,21]. Increasing age is also associated with reduced use of smartphones for internet browsing, social media, and apps [22]. Consequently, mobile app attrition is likely to be increasingly challenging in the population of patients likely to use mobile apps such as CimplyMe; patients with chronic inflammatory diseases are likely to be older and so are likely to be less comfortable using mobile apps. Designing apps with the user in mind and assessing usability in human factors studies will help ensure successful patient engagement.

### Study Limitations

Human factors studies only simulate use in an everyday environment. Patients were asked to behave in the way they thought they would if the scenario was real; however, this may be difficult to judge accurately. Each session was limited to 105 minutes, and, as a result, patients may have felt under some time pressure to complete each task. Time constraints also restricted patients’ ability to familiarize themselves with the app. Additionally, each task was completed discretely, which may have interfered with their familiarization of the app, owing to the lack of continuity between tasks. Patients were selected based on their willingness to use mobile apps, which may limit the generalizability of the results; however, the app will be provided as an additional support component and so use by patients will be voluntary. Therefore, the study population is reflective of the final population of patients who would likely use this in clinical practice. Finally, the number of patients participating in the study was small; therefore, the results may not represent the app’s usability in a wider patient group or capture all potential use errors and difficulties when using the app.

### Conclusions

In a simulated setting, patients were able to use the companion app successfully. No critical task failures were observed; all patients were able to successfully complete both critical and noncritical tasks within two attempts without any formal training. This study demonstrates that the companion app UI is generally intuitive and easy to use among patients familiar with using apps. The app could support CZP patients to self-manage their treatment.

## Appendix

Multimedia Appendix 1 Reasons for critical task difficulties and use errors. Only 12 patients completed critical task 6 (“log an injection date”) due to time constraints.

## Abbreviations

CZP: certolizumab pegol
e-Device: electronic device
EU: European Union
IFU: instructions for use
RA: rheumatoid arthritis
TNF: tumor necrosis factor
UI: user interface
