# Supplementary figures and images for: A Companion App to Support Rheumatology Patients Treated with Certolizumab Pegol: Results From a Usability Study
Source: JMIR Form Res. 2020 Jul 27;4(7):e17373. doi: 10.2196/17373 (PMC7418011; doi:10.2196/17373)

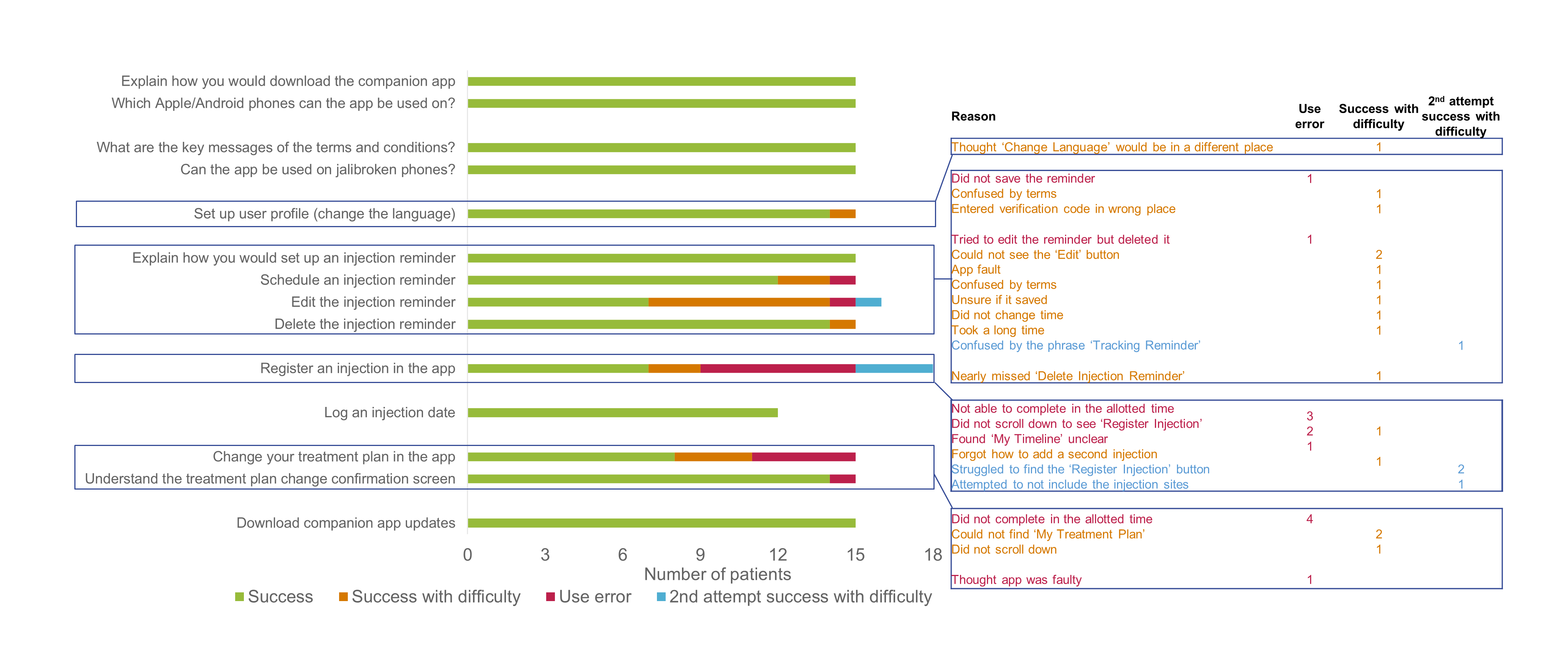

Supplement: Multimedia Appendix 1 [file formative_v4i7e17373_app1.png]
